# Early influence of endotamponade on corneal biomechanical parameters, central corneal thickness and accuracy of intraocular pressure measurement

**DOI:** 10.1038/s41598-023-27407-8

**Published:** 2023-01-04

**Authors:** Zofia Pniakowska, Piotr Jurowski

**Affiliations:** Department of Ophthalmology and Vision Rehabilitation, Medical University of Lodz, Zeromskiego 113, 90-549 Lodz, Poland

**Keywords:** Glaucoma, Corneal diseases, Retinal diseases

## Abstract

To define the influence of air, SF6 gas and silicon oil 1000cs tamponade injection and oil tamponade removal on corneal biomechanics, central corneal thickness and intraocular pressure. 77 eyes referred to vitrectomy were divided into 4 groups: 19 to air tamponade, 21 to SF6 tamponade, 19 to oil tamponade, 18 to oil tamponade removal. Pre- and postoperative corneal hysteresis, corneal resistance factor, corneal thickness, Goldman intraocular pressure (GAT) and corneal compensated intraocular pressure (IOPcc) were analysed. GAT and IOPcc did not change after the air or SF6 tamponade. The oil tamponade injection caused increase in GAT and IOPcc, while the oil removal caused reduction in those parameters. In all groups, preoperative and postoperative values of GAT and IOPcc did not differ. There was no change in corneal thickness and biomechanics after air, SF6 or oil tamponade while after removal of oil those parameters are decreased. The air, SF6 and oil tamponade does not change the corneal thickness and corneal biomechanics. The removal of oil causes decrease in corneal thickness and biomechanics which can lead to bias in intraocular pressure measurement. GAT and IOPcc did not differ in eyes pre- and post-vitrectomy, being similarly reliable measure.

## Introduction

Pars plana vitrectomy (PPV) is a common surgical procedure currently used for a huge spectrum of vitreal and retinal diseases. The use of intraocular air, gas or silicone oil tamponade increased the efficiency and safety of the PPV surgery.

On the other hand, it is well known that the presence of vitreous substitutes carry a risk of some early or late postoperative complications. The former are mostly associated with an increase in intraocular pressure (IOP) and glaucoma due to not fully controlled gas tamponade expansion. Late postoperative complications usually occur in eyes with long-lasting silicone oil tamponade and the chronically elevated IOP as a result of overfilling with silicon oil or a presence of emulsified silicon oil droplets in the anterior chamber. Both mechanisms can lead to blockage of the trabecular meshwork outflow or iridocorneal angle closure. On the other hand, gas or silicon oil tamponade can potentially alter the viscoelasticity and stiffness of the cornea described by corneal biomechanical parameters e.g. corneal hysteresis (CH) and corneal resistance factor (CRF) what in turn is the reason of difficulty in precise and reliable monitoring of IOP^1^. Additionally, the postoperative change in central corneal thickness (CCT) can also influence the reliable IOP measurement in these patients. The corneal response technology, currently used in clinical tonometry, enables the assessment of corneal biomechanical properties as: corneal hysteresis (CH) and corneal resistance factor (CRF), potentially improving the accuracy of the IOP measurement.

The aim of this retrospective study was to define the influence of air, sulfur hexafluoride (SF6) expanding gas, silicon oil 1000cs tamponade administration and removal on corneal biomechanical parameters, CCT and the IOP measurement.

## Methods

The retrospective research was performed in the Department of Ophthalmology and Vision Rehabilitation of University Clinical Hospital in Lodz-Poland. The analyzed medical data was collected between 2019 and 2021. The inclusion criteria for the study comprised: referral to the PPV surgery for following selected indications: rhegmatogenous retinal detachment (RRD), macular hole as well as planned silicon oil tamponade removal. All the patients enrolled for the study were pseudophakic in the studied eye. The study was approved by the Bioethics Committee at the Medical University of Lodz. All methods were performed in accordance with the guidelines and regulations of the Declaration of Helsinki. All patients enrolled in the study had previously signed a voluntary informed consent to the treatment and use of the results of ocular biometry, biomechanics and IOP measurements in future retrospective studies.

The exclusion criteria were as follows: the history of any corneal disease (infective or autoimmune keratitis, corneal trauma, ulceration, corneal scars, any corneal dystrophy, keratoconus, pellucid marginal degeneration, keratoglobus), history of corneal refractive surgery, cataract surgery history less than 3 months, active or history for intraocular inflammation, primary or secondary glaucoma, inadequate lid closure, diabetes mellitus with any ocular complications of diabetes.

After the inclusion process, the medical data of patients were divided into four study groups. All patients treated for macular hole underwent 23 gauge (G) PPV followed by intravitreal air or SF6 gas injection. The latter was chosen based on the size of the macular hole, according to the Modified Gass classification. The first group comprised 19 eyes with macular hole (MH) at the stage 1a—(3 cases), 1b—(5 cases) and stage 2—MH ≤ 400 µm (11 cases) which were referred to PPV with intravitreal air injection. The second ōstudy group consisted of 21 eyes with MH at the stage 3 (MH > 400 µm) and stage 4 (MH > 400 µm with a complete posterior vitreous detachment). Those patients (2nd study group) were managed by PPV with the inverted inner limiting membrane (ILM) flap technique with SF6 tamponade. The concentration of SF6 gas that we used was 25% in all treated eyes. The third study group included 19 eyes with rhegmatogenous retinal detachment (RRD) in whom the PPV with 1000cs silicone oil tamponade was performed. The fourth group was composed of 18 eyes planned to silicon oil 1000cs tamponade removal of at least 6-months duration. In 6 patients with silicone oil tamponade lasting more than 9 months, we observed an emulsification of silicone oil. The group 4 consisted of independent cases. None of the patients in group 3 was later included in group 4.

Medical data of enrolled patients included the pre-operative slit lamp examination and funduscopy. Swept source OCT was performed 7 days prior the surgery to visualize the macular region and classify patients to the 1st and 2nd study group. The measurement of corneal biomechanical properties, IOP and CCT were taken the day before the surgery and on the 1st and 7th day after the surgery. The ocular response analyzer (ORA) provided results of CH, CRF and corneal compensated intraocular pressure (IOPcc). Additionally, for each measurement, the device estimated the value of the waveform score (WS), which defines the quality of the measurement based on the symmetry of applanation related to the condition of the patient's cornea. WS values ​​were presented on a scale from zero (0) to ten (10). The Goldmann applanation tonometry (GAT) was used for IOP assessment. CCT was measured by Tomey Optical Biometer OA-2000.

The distribution of all measurable variables was tested using the Shapiro–Wilk test. All data showed a normal distribution and were presented as mean and standard deviation (SD). The Student’s t-test for dependent samples was used to compare pre- and postoperative values of the same parameter in the same study group. The impact between two variables was assessed by the coefficient of determination, which is the square of the correlation coefficient. The differences between the mean values of parameters were considered as a statistical significant, where the p value was less than 0.05. The statistical analysis was performed using Statistica 12.

### Ethical approval

The study was approved by the Bioethics Committee at the Medical University of Lodz. All patients enrolled in the study had previously signed a voluntary informed consent to the use of their medical data in the retrospective study.

## Results

**Figure 1:**
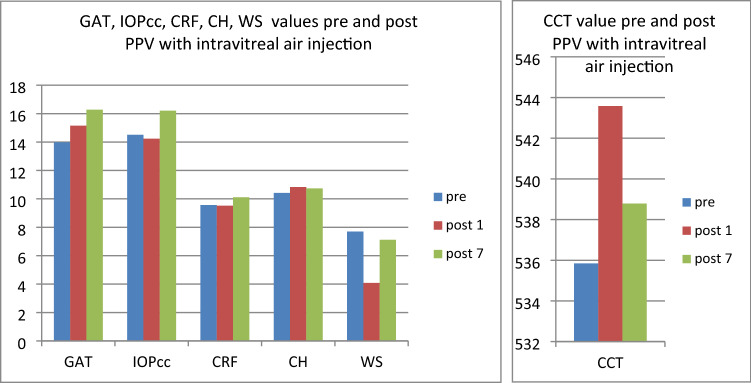
Plot of mean pre and postoperative values of *GAT* Goldman intraocular pressure, *IOPcc* corneal compensated intraocular pressure, *CRF* corneal resistance factor, *CH* corneal hysteresis, *WS* waveform score, *CCT* central corneal thickness in the first study group—patients with MH referred to PPV with intravitreal air injection.

In the first study group (patients referred to PPV with intravitreal air application) there were statistically significant differences in mean values of: CCT (t = − 3.11, p < 0.05) and WS (t = 5.63, p < 0.05) when compared the pre and postoperative measurement taken on 1st postoperative day (Fig. 1).

**Figure 2:**
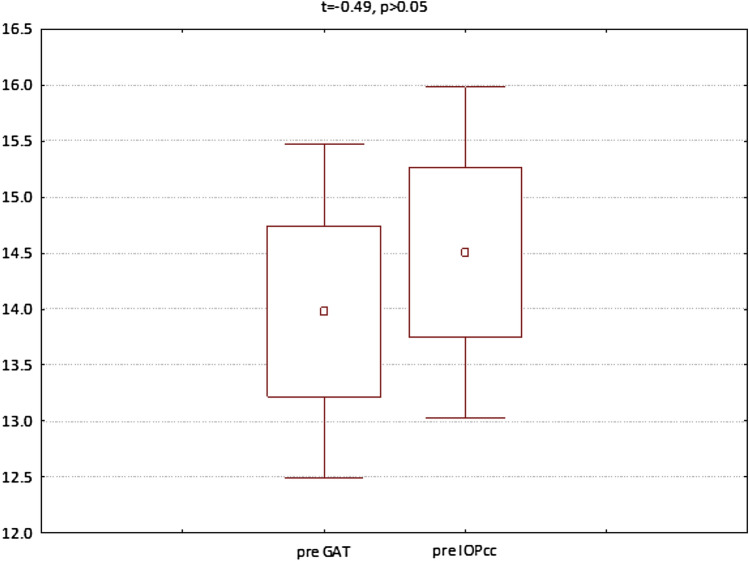
Preoperative GAT and IOPcc values distribution and differences in patients referred to PPV with air injection.

**Figure 3:**
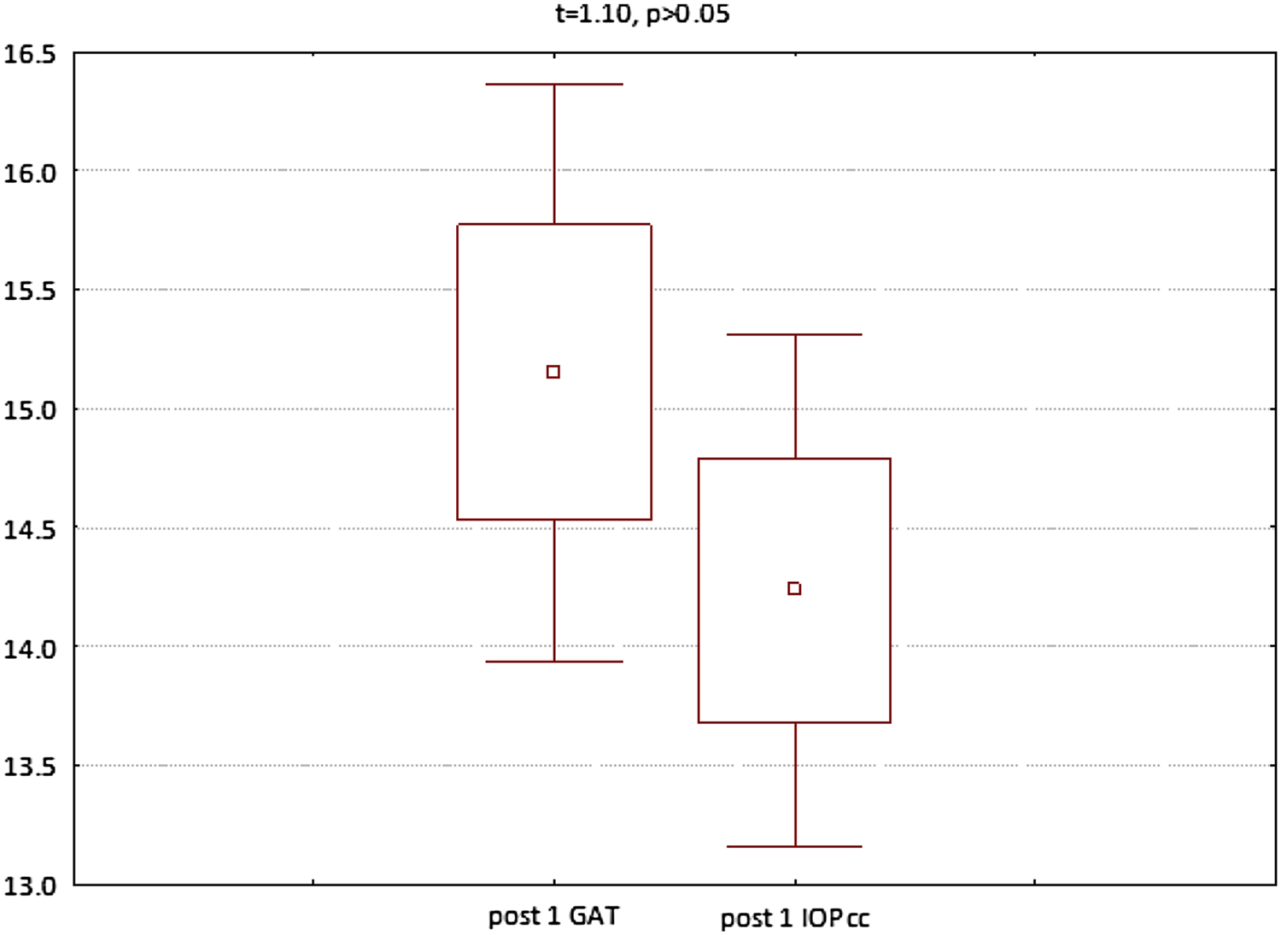
GAT and IOPcc values distribution and differences on the 1st postoperative day in patients referred to PPV with air injection.

**Figure 4:**
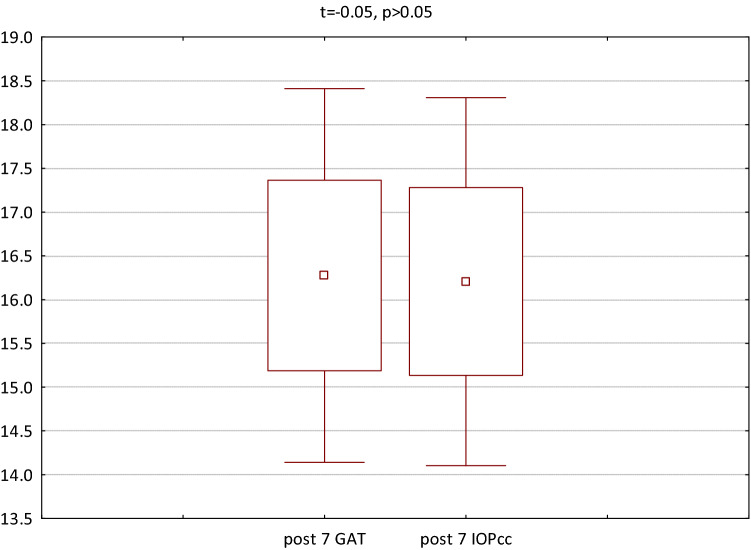
GAT and IOPcc values distribution and differences on the 7th postoperative day in patients referred to PPV with air injection.

The GAT and IOPcc values distribution and differences measured preoperatively and on the 1st and 7th postoperative day were demonstrated in the Figs. 2, 3 and 4. GAT correlated strongly positively with IOPcc values in preoperative (r2 = 0.83, p < 0.05) and on 1st and 7th postoperative measurement (respectively: r2 = 0.67, p < 0.05; r2 = 0.69, p < 0.05). The strong positive correlation was also observed between CH and CRF when analysed separately pre and postoperatively on 1st and 7th day (respectively: r2 = 0.87, p < 0.05; r2 = 0.69, p < 0.05; r2 = 0.37, p < 0.05). Preoperative values of CCT correlated positively with CRF (r2 = 0.34, p < 0.05) and with CH (r2 = 0.27, p < 0.05) (Table 1). CCT and CRF correlated positively when measured on 7th postoperative day (r2 = 0.37, p < 0.05) (Table 1).

**Table 1:** Correlations of selected parameters assessed pre and postoperatively in the first study group—patients with MH referred to PPV with intravitreal air injection (*GAT*—Goldman intraocular pressure, *IOPcc*- corneal compensated intraocular pressure, *CH*- corneal hysteresis *CRF*- corneal resistance factor, *CCT*- central corneal thickness, *pre*- measured on the 1^st^ preoperative day, *post 1*—measured on the 1^st^ postoperative day, *post 7*—measured on the 7th postoperative day).

	r(X,Y)	r2	t	p
pre GAT/pre IOPcc	0.911755	0.831298	9.152565	0.000000
post 1 GAT/post 1 IOPcc	0.821416	0.674725	5.93831	0.000016
post 7 GAT/post 7 IOPcc	0.832116	0.692417	6.186246	0.000010
pre CRF/pre CH	0.933571	0.871555	10.74023	0.000000
post 1 CRF/post 1 CH	0.833970	0.695507	6.23141	0.000009
post 7 CRF/post 7 CH	0.612985	0.375751	3.198860	0.005260
pre CRF/pre CCT	0.583033	0.339927	2.958840	0.008793
post 1 CRF/post 1 CCT	0.290941	0.084647	1.253821	0.226876
post 7 CRF/post 7 CCT	0.612697	0.375397	3.196450	0.005288
pre CH/pre CCT	0.521593	0.272059	2.520622	0.021998
post 1 CH/post 1 CCT	0.092565	0.008568	0.383303	0.706246
post 7 CH/post 7 CCT	0.432384	0.186956	1.977139	0.064475

**Figure 5:**
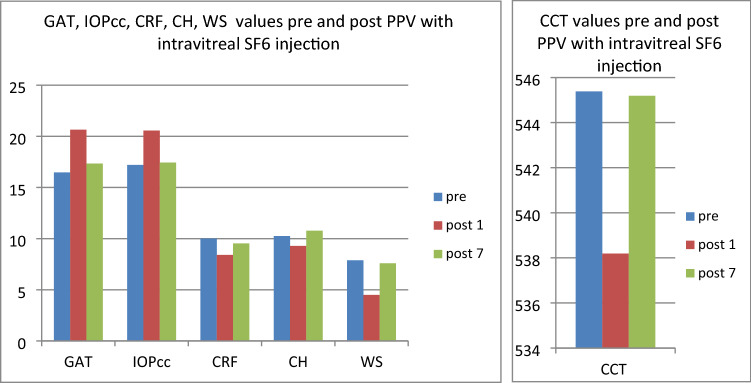
Plot of mean pre and postoperative values of GAT—Goldman intraocular pressure, IOPcc- corneal compensated intraocular pressure, CRF- corneal resistance factor, CH- corneal hysteresis, WS- waveform score, CCT- central corneal thickness in the second study group—patients with MH referred to PPV with intravitreal SF6 gas injection.

**Figure 6:**
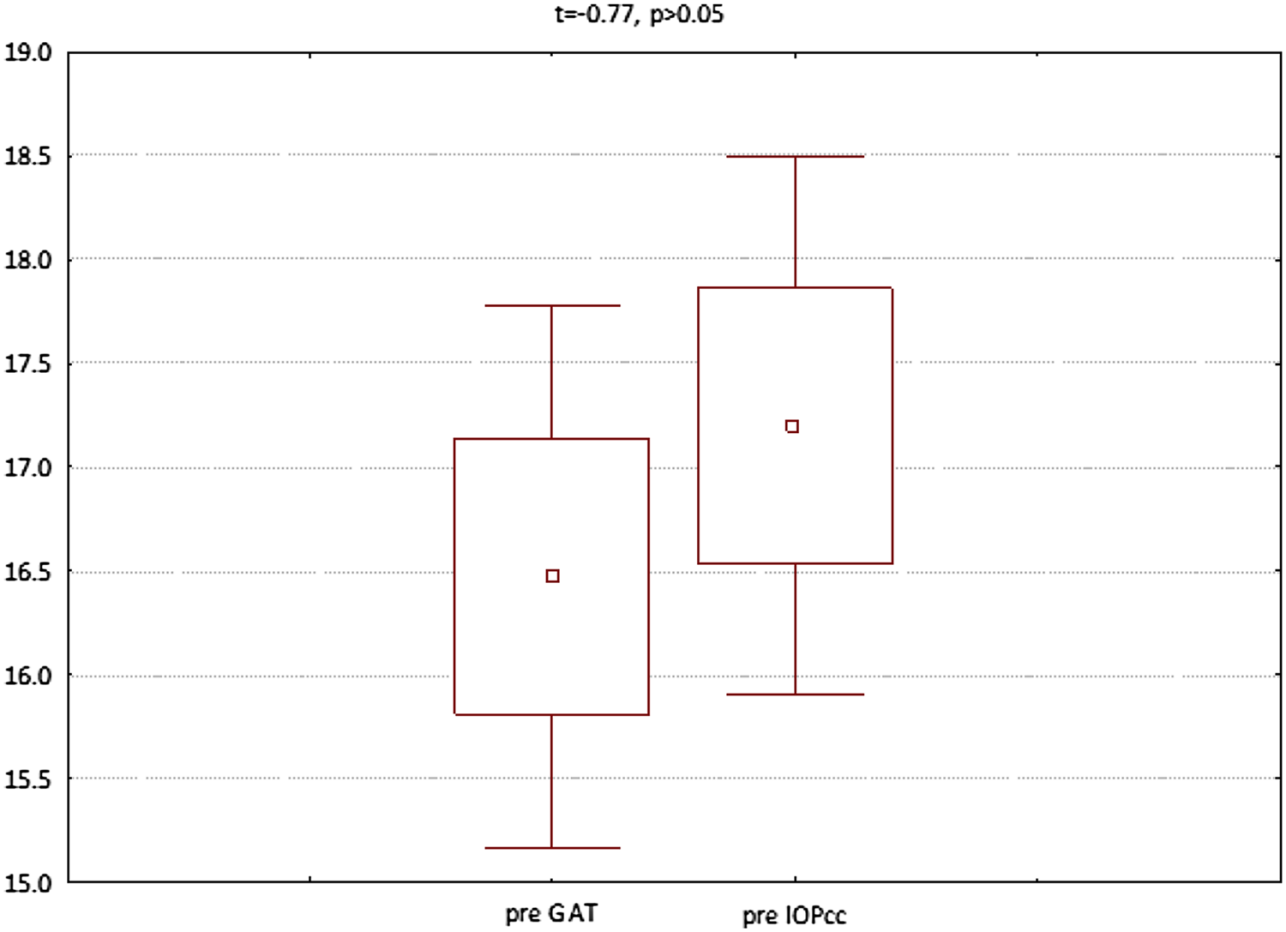
Preoperative GAT and IOPcc values distribution and differences in patients referred to PPV with SF6 gas injection.

**Figure 7:**
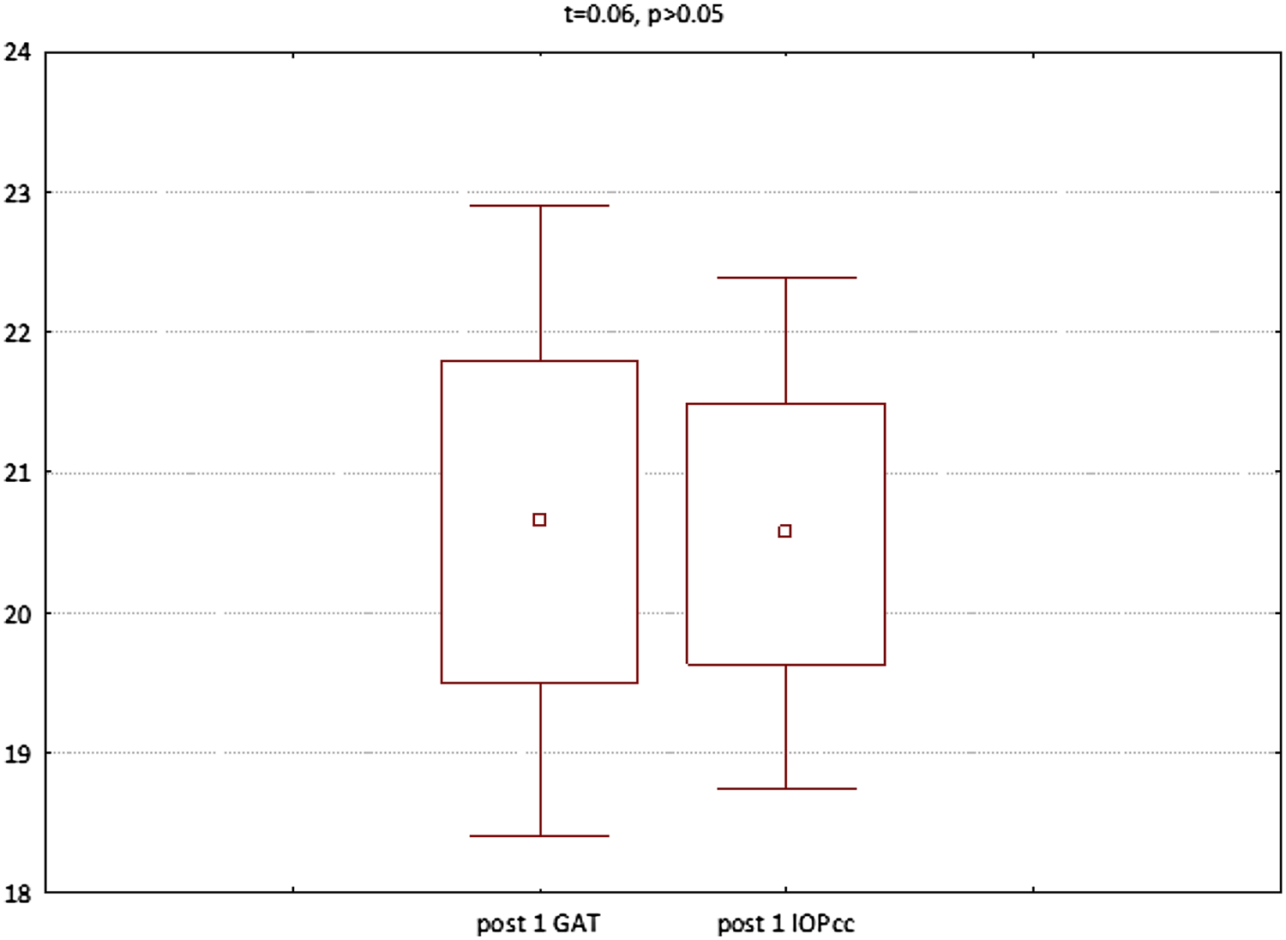
GAT and IOPcc values distribution and differences on the 1st postoperative day in patients referred to PPV with SF6 gas injection.

**Figure 8:**
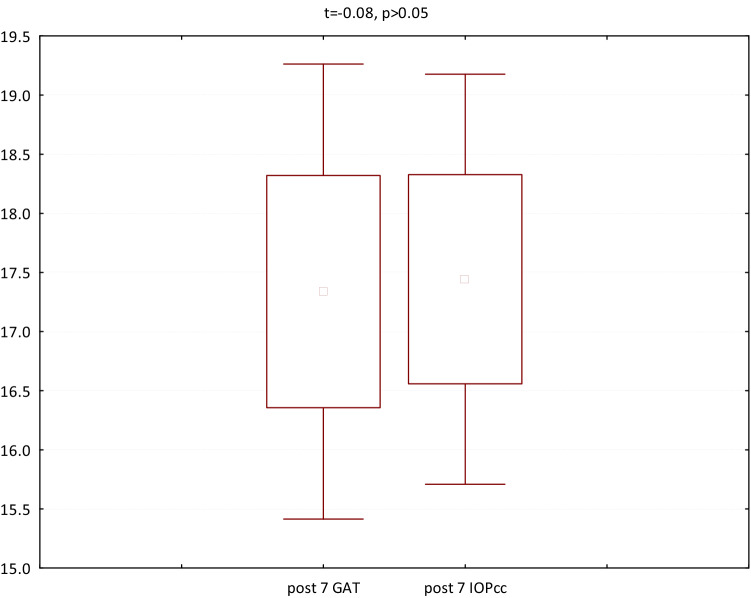
GAT and IOPcc values distribution and differences on the 7th postoperative day in patients referred to PPV with SF6 gas injection.

In the second group (patients referred to PPV with intravitreal SF6 gas injection) both GAT (t = − 3.06, p < 0.05) and IOPcc (t = − 2.97, p < 0.05) were significantly increased on 1st postoperative day. In turn, all the following parameters were significantly decreased on 1st postoperative morning: CCT (t = 4.71, p < 0.05), CH (t = 2.97, p < 0.05), CRF (t = 5.82, p < 0.05), WS (t = 10.76, p < 0.05) (Fig. 5). The values of GAT and IOPcc as well as differences in those parameters were presented in Figs. 6, 7 and 8. The GAT values correlated positively with IOPcc in preoperative (r2 = 0.88, p < 0.05) and postoperative measurement on 1st and 7th day (respectively r2 = 0.92, p < 0.05; r2 = 0.83, p < 0.05). Similarly, there was a strong positive correlation of CH and CRF both in pre- (r2 = 0.84, p < 0.05) and in both postoperative measurements on: 1st day (r2 = 0.19, p < 0.05) and 7th day (r2 = 0.22, p < 0.05). Preoperative CCT correlated positively with preoperative CRF (r2 = 0.66, p < 0.05) and CH (r2 = 0.55, p < 0.05). There was also a positive correlation between CCT and CRF both on 1st (r2 = 0.25, p < 0.05) and 7th (r2 = 0.65, p < 0.05) postoperative day (Table 2).

**Table 2:** Correlations of selected parameters assessed pre and postoperatively in the second study group—patients with MH referred to PPV with intravitreal SF6 gas injection (*GAT* Goldman intraocular pressure, *IOPcc* corneal compensated intraocular pressure, *CH* corneal hysteresis, *CRF* corneal resistance factor, *CCT* central corneal thickness, *pre* measured on the 1st preoperative day, *post 1* measured on the 1st postoperative day, *post 7* measured on the 7th postoperative day).

	r(X,Y)	r2	t	p
pre GAT/pre IOPcc	0.941417	0.886265	12.16779	0.000000
post 1 GAT/post 1 IOPcc	0.958168	0.918087	14.59288	0.000000
post 7 GAT/post 7 IOPcc	0.911841	0.831455	9.681394	0.000000
pre CRF/pre CH	0.915822	0.838730	9.940569	0.000000
post 1 CRF/post 1 CH	0.434869	0.189111	2.105014	0.048827
post 7 CRF/post 7 CH	0.470696	0.221555	2.325431	0.031276
pre CRF/pre CCT	0.813825	0.662311	6.104489	0.000007
post 1 CRF/post 1 CCT	0.498278	0.248281	2.505078	0.021509
post 7 CRF/post 7 CCT	0.810621	0.657106	6.034126	0.000008
pre CH/pre CCT	0.746650	0.557486	4.892489	0.000101
post 1 CH/post 1 CCT	0.299566	0.089740	1.368630	0.187081
post 7 CH/post 7 CCT	0.370102	0.136975	1.736546	0.098654

**Figure 9:**
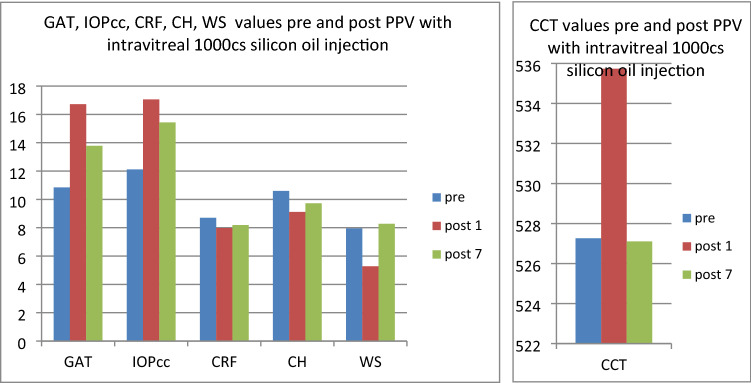
Plot of mean pre and postoperative values of *GAT* Goldman intraocular pressure, *IOPcc* corneal compensated intraocular pressure, *CRF* corneal resistance factor, *CH* corneal hysteresis, *WS* waveform score, *CCT* central corneal thickness in the third study group—patients with RRD qualified for PPV with 1000cs silicone oil injection.

**Figure 10:**
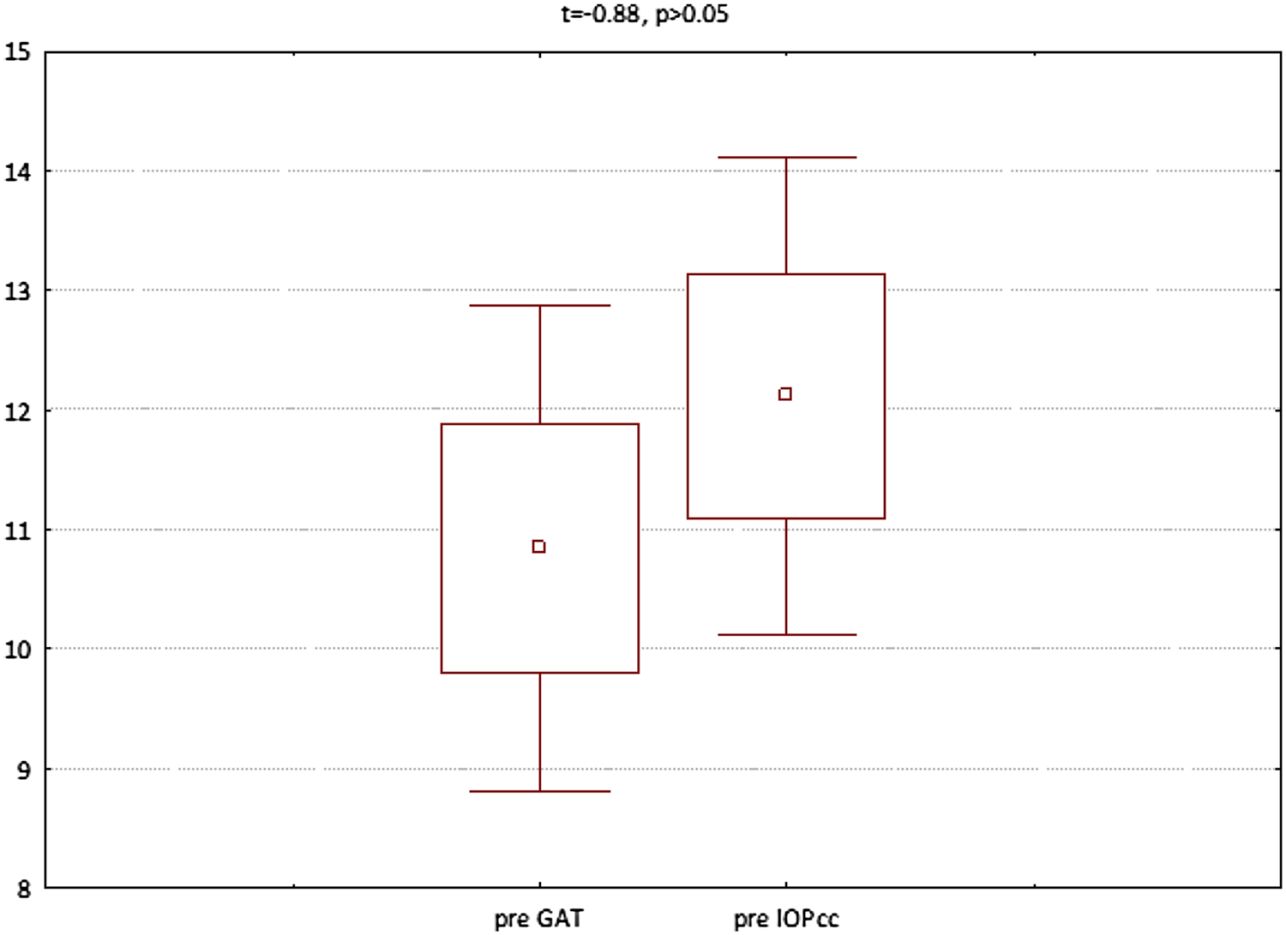
Preoperative GAT and IOPcc values distribution and differences in patients referred to PPV with silicon oil 1000cs injection.

**Figure 11:**
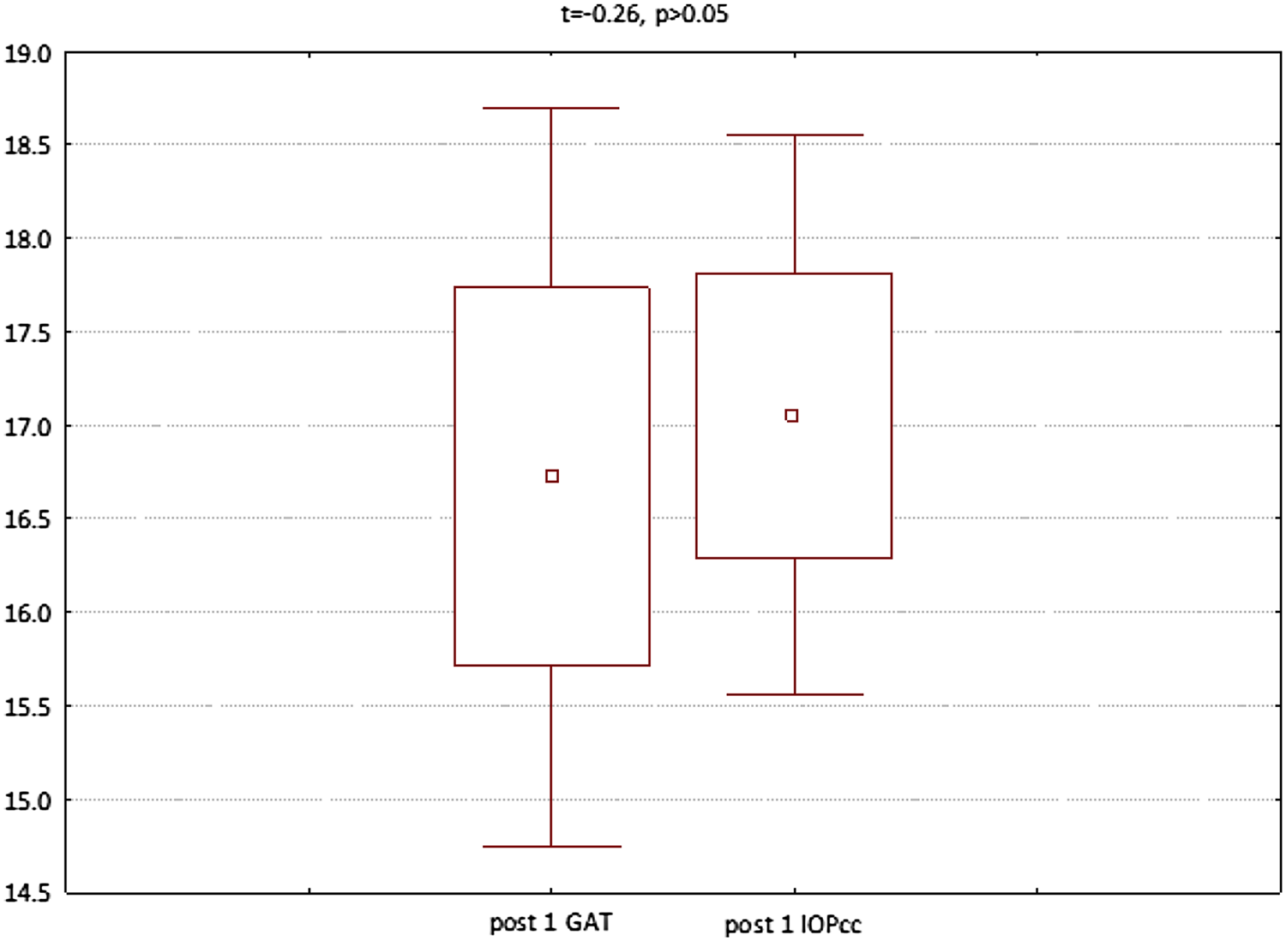
GAT and IOPcc values distribution and differences on the 1st postoperative day in patients referred to PPV with silicon oil 1000cs injection.

**Figure 12:**
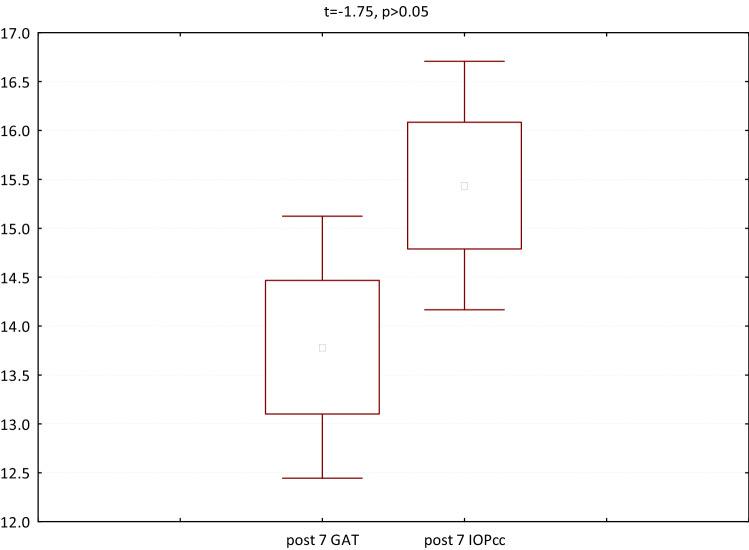
GAT and IOPcc values distribution and differences on the 7th postoperative day in patients referred to PPV with silicon oil 1000cs injection.

In the third group (patients referred to PPV with silicone oil 1000cs administration) both GAT and IOPcc were significantly elevated on 1st postoperative day respectively: GAT (t = − 5.48, p < 0.05) and IOPcc (t = − 5.47 p < 0.05). Similarly, GAT (t = − 2.85, p < 0.05) and IOPcc (t = − 3.08, p < 0.05) were significantly elevated on the 7th postoperative day in comparison to the preoperative values. CH values were significantly decreased on 7th postoperative day (t = 2.39, p < 0.05). In turn, there was a significant rise in postoperative CCT (t = − 9.82, p < 0.05) and WS (t = 4.66 p < 0.05) on 1st postoperative day (Fig. 9). The mean values and differences between GAT and IOPcc have been included in Figs. 10, 11, and 12. The values of GAT and IOPcc correlated positively with each other in preoperative (r2 = 0.85, p < 0.05) and two postoperative assessments on 1st and 7th day (respectively: r2 = 0.79, p < 0.05; r2 = 0.67, p < 0.05). There was a strong positive correlation between CH and CRF when measured pre (r2 = 0.83, p < 0.05), 1st day postoperatively (r2 = 0.86, p < 0.05) and 7th day following the surgery (r2 = 0.48, p < 0.05). Similarly, preoperative values of CCT correlated positively with preoperative CRF (r2 = 0.81, p < 0.05) and CH (r2 = 0.75, p < 0.05). In addition, there was a positive correlation between postoperative CCT and CRF values measured on 1st and 7th postoperative day respectively: (r2 = 0.55, p < 0.05; r2 = 0.86, p < 0.05) and CH respectively: (r2 = 0.46, p < 0.05; r2 = 0.66, p < 0.05) (Table 3).

**Table 3:** Correlations of selected parameters assessed pre and postoperatively in the third study group—patients with RRD qualified for PPV with 1000cs silicone oil injection (*GAT* Goldman intraocular pressure, *IOPcc*- corneal compensated intraocular pressure, *CH* corneal hysteresis, *CRF* corneal resistance factor, *CCT* central corneal thickness, *pre* measured on the 1st preoperative day, *post 1* measured on the 1st postoperative day, *post* *7* measured on the 7th postoperative day).

	r(X,Y)	r2	t	p
pre GAT/pre IOPcc	0.924587	0.854862	10.00649	0.000000
post 1 GAT/post 1 IOPcc	0.890361	0.792743	8.06373	0.000000
post 7 GAT/post 7 IOPcc	0.817838	0.668859	5.859836	0.000019
pre CRF/pre CH	0.912911	0.833406	9.221953	0.000000
post 1 CRF/post 1 CH	0.931572	0.867826	10.56497	0.000000
post 7 CRF/post 7 CH	0.695781	0.484111	3.994099	0.000939
pre CRF/pre CCT	0.903014	0.815434	8.666481	0.000000
post 1 CRF/post 1 CCT	0.745001	0.555026	4.604835	0.000252
post 7 CRF/post 7 CCT	0.927744	0.860709	10.24923	0.000000
pre CH/pre CCT	0.867126	0.751908	7.177950	0.000002
post 1 CH/post 1 CCT	0.677781	0.459387	3.800762	0.001428
post 7 CH/post 7 CCT	0.814965	0.664168	5.798317	0.000021

**Figure 13:**
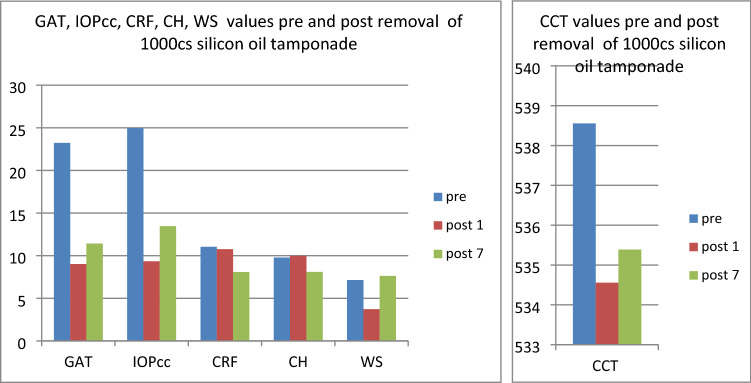
Plot of mean pre and postoperative values of *GAT* Goldman intraocular pressure, *IOPcc* corneal compensated intraocular pressure, *CRF* corneal resistance factor, *CH* corneal hysteresis, *WS* waveform score, *CCT* central corneal thickness in the fourth group-patients referred to the removal of 1000cs silicon oil tamponade.

**Figure 14:**
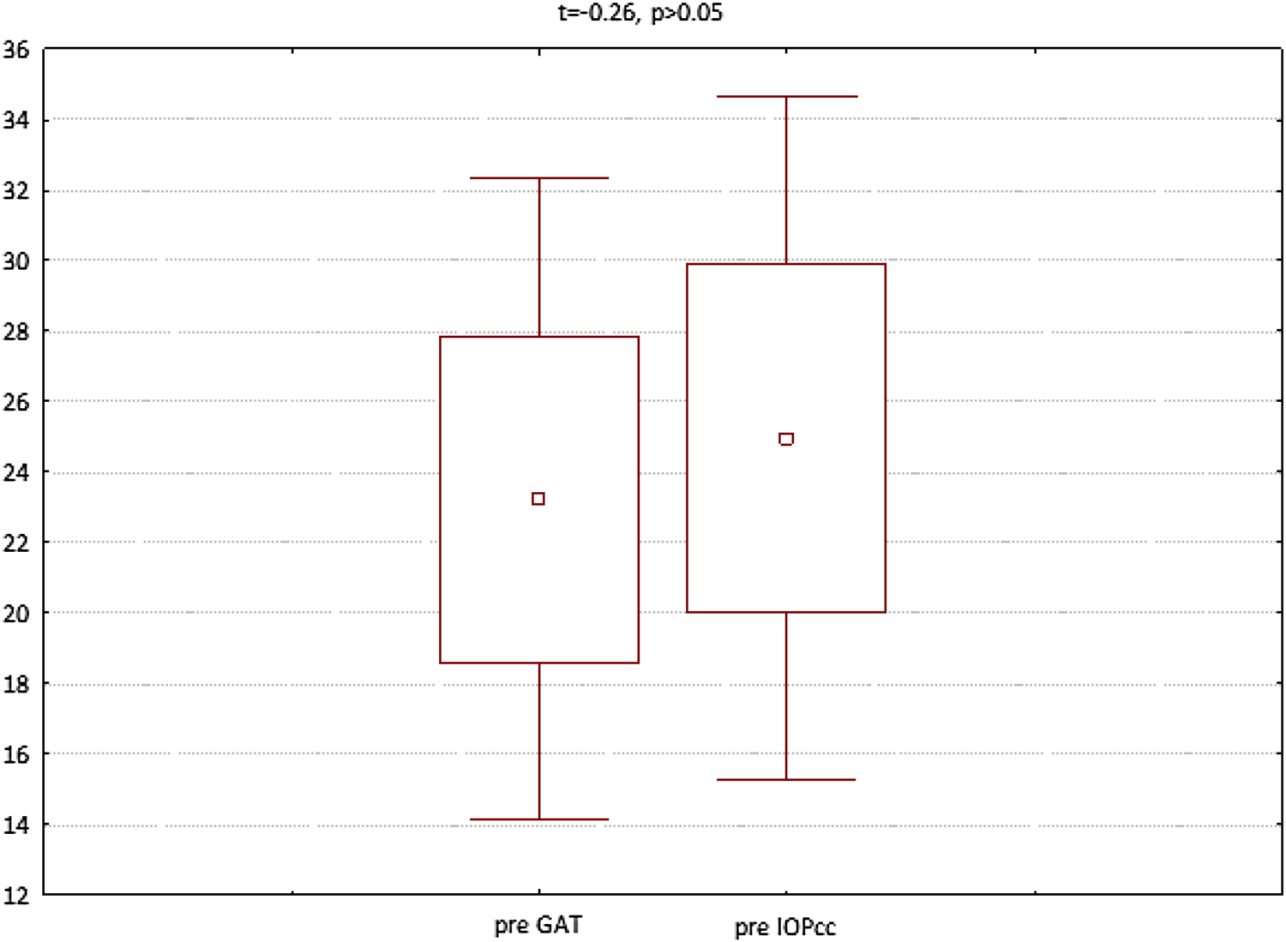
Preoperative GAT and IOPcc values distribution and differences in patients referred to silicon oil tamponade removal.

**Figure 15:**
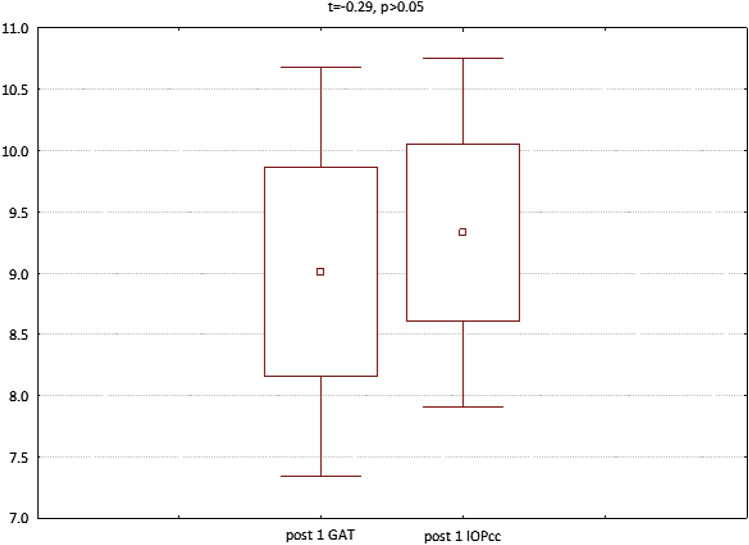
GAT and IOPcc values distribution and differences on the 1st postoperative day in patients referred to silicon oil tamponade removal.

**Figure 16:**
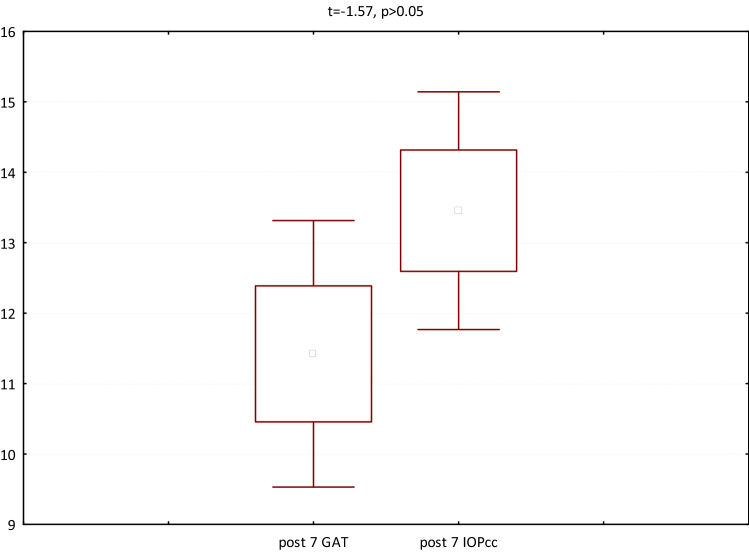
GAT and IOPcc values distribution and differences on the 7th postoperative day in patients referred to silicon oil tamponade removal.

In the fourth group (patients referred for the removal of silicone oil tamponade), GAT (t = 3.23, p < 0.05), IOPcc (t = 3.32, p < 0.05) and WS (t = 7.79, p < 0.05) were significantly reduced on 1st postoperative day. In turn, when observed on the 7th day after the surgical procedure, the values of CCT (t = 2.77, p < 0.05), GAT (t = 2.56, p < 0.05), IOPcc (t = 2.44, p < 0.05), CH (t = 2.86, p < 0.05) and CRF (t = 4.67, p < 0.05) were significantly reduced in comparison to the preoperative values (Fig. 13). The values of GAT and IOPcc and differences between those parameters at each observation point were presented in Figs. 14, 15 and 16. GAT and IOPcc parameters correlated positively in preoperative (r2 = 0.99, p < 0.05) and postoperative assessment on 1st (r2 = 0.89, p < 0.05) and 7th day (r2 = 0.66, p < 0.05) after the surgery. CCT values correlated positively with CRF in preoperative assessment (r2 = 0.70, p < 0.05), as well as in postoperative measurement taken on 1st (r2 = 0.70, p < 0.05) and 7th (r2 = 0.50, p < 0.05) day (Table 4).

**Table 4:** Correlations of selected parameters assessed pre and postoperatively in the fourth group-patients referred to the removal of 1000cs silicon oil tamponade (*GAT* Goldman intraocular pressure, *IOPcc* corneal compensated intraocular pressure, *CH* corneal, *CRF* corneal resistance factor, *CCT* central corneal thickness, *pre* measured on the 1st preoperative day, *post 1* measured on the 1st postoperative day, *post 7* measured on the 7th postoperative day).

	r(X,Y)	r2	t	p
pre GAT/pre IOPcc	0.996890	0.993789	50.59837	0.000000
post 1 GAT/post 1 IOPcc	0.942866	0.888996	11.31985	0.000000
post 7 GAT/post 7 IOPcc	0.814066	0.662704	5.606787	0.000039
pre CRF/pre CH	0.162952	0.026553	− 0.660638	0.518244
post 1 CRF/post 1CH	− 0.142614	0.020339	− 0.57635	0.572401
post 7 CRF/post 7 CH	− 0.464773	0.216014	− 2.09965	0.051975
pre CRF/pre CCT	0.838493	0.703071	6.155073	0.000014
post 1 CRF/post 1 CCT	0.834787	0.696870	6.064864	0.000016
post 7 CRF/post 7 CCT	0.709387	0.503229	4.025918	0.000977
pre CH A/pre CCT	− 0.201949	0.040783	− 0.824789	0.421618
post 1 CH/post 1 CCT	− 0.190207	0.036179	− 0.774974	0.449657
post 7 CH/post 7 CCT	− 0.185797	0.034520	− 0.756357	0.460428

## Discussion

The common use of vitreous substitutes such as: air, gas (SF6, C3F8), silicon oil (1000cs, 2000cs, 5000cs) tamponade improve the PPV outcomes, however it is also related to some postoperative complications. One of the most severe complications following the PPV surgery with tamponade injection is increase in IOP, which can lead to mechanical compression and blood flow disturbances within the optic nerve head. The damage to retinal nerve fiber layer (RNFL) and ganglion cell complex (GCC) results in irreversible glaucomatous changes followed by visual field defects^2–5^. In addition, the use of intravitreal substitutes can cause corneal changes such as: loss of corneal endothelial cells, corneal decompensation, band keratopathy and can induce postoperative changes in CCT and corneal biomechanical parameters as CH and CRF^6–11^. This changes, in turn, influence the accurate IOP measurement even with the leading tonometry methods.

In the study of Muether et al., in eyes after PPV with SF6 tamponade, the early significant rise in GAT (measured on the first postoperative morning) was noted in 37.9% of cases^12^. Our results strongly confirmed the Muether's reports, as there was significant rise in GAT and IOPcc on 1st postoperative day in patients with SF6 tamponade when compared to preoperative measurements. In contrast, in the group where the air tamponade was applied, there was no significant difference in GAT or IOPcc when measured on the 1st day after the surgery. In the study of Teke M, who compared IOP values measured with ORA (IOPg and IOPcc) before and 1 week after the PPV with SF6 or C3F8 injection, there was significant rise of IOPcc and IOPg in group with long acting C3F8 gas, with no significant change of IOPcc and IOPg values in SF6 group^7^. This is consistent with our results, as there was no significant rise in GAT and IOPcc in both groups of patients, who underwent PPV with intravitreal air or SF6 injection when measured on 7th postoperative day. It is thought that the reason for lack of change in IOP in patients with air or SF6 injection 7 days after the surgery is lack of air expansile properties and slightly expansile concentration of SF6 (25%) which does not elevate IOP significantly.

The recent clinical studies suggest the significant rise in IOP after PPV with silicon oil tamponade^3,10,13,14^. The mechanism of intraocular elevation is explained by various mechanisms: oil-induced intraocular inflammation, mechanical obstruction of trabecular meshwork by oil droplets, anterior displacement of irido-lenticular diaphragm with consecutive anterior chamber angle narrowing, pupillary block with silicon oil displacement to the anterior chamber, prone head position in the early postoperative period^3,10,13,14^. Similarly, our results showed a significant rise in GAT and IOPcc values in patients who underwent PPV with silicone oil tamponade, when measured on the 1st and 7th postoperative day in comparison to the preoperative measurement.

In turn, after the removal of silicone oil tamponade, there was observed a significant lowering in both GAT and IOPcc on 1st and 7th postoperative day when compared to preoperative values. These results are consistent with the previous report of Jawad et al., which revealed significantly lowered values of IOP in eyes after removal of silicon oil in comparison with preoperative values^15^. Similarly, Issa et al. observed decrease in IOP in patients after silicon oil tamponade removal^16^. Moreover, lots of authors described significant intraocular hypotony, defined as IOP values of 5 mmHg or less after the silicon oil removal^17–20^. However, early intraocular hypotony was not observed in our study as the mean values of GAT and IOPcc were both above 9.0 mm Hg (respectively: 9.01 ± 3.6 SD, 9.33 ± 3.08 SD) when measured on 1st day postoperatively. In all groups enrolled in our study, the preoperative and postoperative values of GAT and IOPcc did not differ significantly. Moreover, our study showed strong positive correlation between GAT and IOPcc what can suggest that both measurements are similarly reliable in patients who have undergone PPV with tamponade injection or removal.

On the 1st postoperative day, corneal biomechanical parameters as CH and CRF were significantly lowered in patients who underwent PPV with SF6 injection. In the rest of study groups following PPV procedures, CH and CRF did not change significantly on the 1st postoperative day. CCT values were significantly increased on 1st postoperative morning in eyes with air tamponade and oil tamponade in contrast to eyes with SF6 tamponade administered, where CCT was significantly decreased. Following oil tamponade removal, the CCT mean value did not change significantly on the 1st postoperative day. Although the results of measurements on the 1st day after surgery suggest differences in the parameters of the corneal biomechanics, the authors believe that this is the effect of poor reliability of the measurements, as evidenced by a significant reduction in the WS coefficient. WS coefficient (which is an indicator of the quality and reliability of the measurement) was significantly reduced in all groups of patients when measured on the first day after surgery.

In contrast, there were no significant changes of WS values on 7th postoperative day, when compared to the preoperative values in all study groups. This observation may suggest that the measurements of IOP and biomechanical properties of the cornea made on the 7th postoperative day were burdened with a smaller measurement error than those made on the 1st day after PPV.

Our results showed no statistically significant change in corneal parameters including CCT, CH and CRF as assessed on the 7th day after the PPV with air or SF6 tamponade. Similarly, CCT and CRF did not change significantly on the 7th day after silicone oil injection, however the mean value of CH decreased significantly in this group of patients. The lowering of the CH, in turn, can be related to a significant rise in IOPcc and GAT, observed in these patients on 7th postoperative morning. This relationship proved by our study in eyes with silicon oil tamponade was also previously described in some clinical studies over the effect of rise in IOP on CH in glaucomatous eyes^21,22^.

In patients who underwent PPV surgery with removal of silicone oil tamponade, the CCT, CH and CRF values were significantly reduced when measured on 7th day postoperatively in comparison to preoperative values. The question arises, whether the reduction was related to the surgery itself or it was a result of 6-months presence of intraocular silicon oil tamponade or both. Current studies suggest that in eyes with chronically elevated IOP, CCT is significantly reduced, what can be consistent with long-term increase in IOP related to silicon oil tamponade lasting 6-months in our patients^23^. The pathomechanism of CCT changes in patients with elevated IOP is not entirely clear^24^. It is suggested that CCT reduction in glaucomatous eyes may be the effect of remodeling of the corneal stroma under the increased IOP and pressure exerted on stromal collagen fibers^25^. This, in turn, leads to the stretching of the cornea and in consequence its thinning^25^. As it was mentioned above, remodeling of the corneal stroma in response to chronically elevated IOP in case of on silicon oil tamponade can be responsible for the decreased values of CH. According to our results, CRF values were elevated in eyes with silicon oil and have been significantly reduced after its removal. It is suggested that elevated CRF is a derivative of increased corneal stiffness through a transient tension in the stromal collagen fibers in response to an increase in IOP^26^. As we suppose, after the removal of silicone oil and reported significant decrease in IOPcc and GAT, the taut stromal fibers of the cornea relaxed leading to postoperative reduction of CRF.

Our results revealed a strong positive correlation between CH and CRF in all study groups pre and postoperatively, except patients who underwent silicon oil removal. In the other words, the corneal viscoelasticity described by CH correlated with stiffness and total resistance of the cornea described by CRF in patients referred to PPV, as well as in eyes that have undergone PPV with air, SF6 or silicon oil injection. This relationship between CH and CRF was not observed in patients prior, as well as on 7th day after silicon oil tamponade removal. What is more, there was a strong preoperative and postoperative correlation of CCT only with CRF but not with CH. This result is a strong argument that the silicon oil tamponade affects corneal viscoelasticity (CH), its stiffness (CRF) and thickness (CCT) in different ways, and this effect persists even after the removal of tamponade.

The major limitation of our study is the retrospective design. Further prospective clinical trials with longer follow up time and larger sample size are needed to expand the results of the study. Moreover, the duration time of silicone oil tamponade in the fourth study group was at least 6 months, which could have caused structural changes in the studied eyes. The morphological changes could have had an influence on the IOP, corneal biomechanics and CCT values measured, causing bias in the obtained results.

## Conclusions

The PPV with intravitreal air or 25% SF6 injection does not significantly influence GAT and IOPcc, while PPV with silicone oil tamponade procedure causes significant rise in GAT and IOPcc values when measured on the 7th postoperative day. In turn, the removal of silicone oil tamponade causes a significant drop in both GAT and IOPcc. GAT and IOPcc did not differ significantly but correlated positively in pre-and postoperative assessments, which can prove that both measures are similarly reliable in patients after PPV procedure. The PPV with intravitreal air or 25% SF6 injection does not influence CCT and its biomechanical parameters including CH and CRF, when assessed on the 7th postoperative day. Similarly, PPV with silicone oil tamponade does not change the values of CCT and CRF, but it causes a significant decrease in CH. In turn, the PPV with removal of silicone oil tamponade causes significant decrease in CCT, CH and CRF values. Finally, there is a strong positive correlation between CH and CRF in patients who undergone PPV with air, SF6 or silicon oil injection. However, this relationship between CH and CRF was not observed in patients just after the removal of silicon oil tamponade. This result suggests that the chronic oil tamponade decreases corneal viscoelasticity (CH) and its stiffness (CRF) indeed but in a different way as evidenced by the lack of correlation between these parameters. The solution to this problem remains unclear and will become the subject of further research on the impact of intraocular surgical procedures on corneal biomechanics and precise IOP measurement.

## Data Availability

The datasets used and/or analysed during the current study are available from the corresponding author on reasonable request.

## Abbreviations

CCT: Central corneal thickness
IOPcc: Corneal compensated intraocular pressure
CH: Corneal hysteresis
CRF: Corneal resistance factor
GAT: Goldman intraocular pressure
ILM: Inner limiting membrane
MH: Macular hole
PPV: Pars plana vitrectomy
RRD: Rhegmatogenous retinal detachment
SF6: Sulfur hexafluoride
WS: Waveform score
